# The filamentous fungus *Penicillium chrysogenum* analysed via flow cytometry—a fast and statistically sound insight into morphology and viability

**DOI:** 10.1007/s00253-019-09943-4

**Published:** 2019-06-19

**Authors:** Lukas Veiter, Christoph Herwig

**Affiliations:** 1 CD Laboratory on Mechanistic and Physiological Methods for Improved Bioprocesses, Vienna University of Technology, Gumpendorferstrasse 1a/166, 1060 Vienna, Austria; 2 Research Area Biochemical Engineering, Institute of Chemical, Environmental and Bioscience Engineering, Vienna University of Technology, Gumpendorferstrasse 1a/166-4, 1060 Wien, Austria

**Keywords:** Filamentous fungi, *Penicillium chrysogenum*, Flow cytometry, Viability, Morphology, Pellets

## Abstract

Filamentous fungi serve as production host for a number of highly relevant biotechnological products, like penicillin. In submerged culture, morphology can be exceptionally diverse and is influenced by several process parameters, like aeration, agitation, medium composition or growth rate. Fungal growth leads to several morphological classes encompassing homogeneously dispersed hyphae and various forms of hyphal agglomerates and/or clump structures. Eventually, the so-called pellet structure can be formed, which represents a hyphal agglomerate with a dense core. Pellet structures can hinder oxygen and substrate transport, resulting in different states of viability, which in turn affects productivity and process control. Over the years, several publications have dealt with methods to either gain morphological insight into pellet structure or determine biomass viability. Within this contribution, we present a way to combine both in a flow cytometry–based method employing fluorescent staining. Thereby, we can assess filamentous biomass in a statistically sound way according to (i) morphology and (ii) viability of each detected morphological form. We are confident that this method can shed light on the complex relationship between fungal morphology, viability and productivity—in both process development and routine manufacturing processes.

**Electronic supplementary material:**

The online version of this article (10.1007/s00253-019-09943-4) contains supplementary material, which is available to authorized users.

## Introduction

Successful cultivation strategies involving filamentous fungi need to consider the organism’s morphology. For example, *Penicillium chrysogenum* comprises several morphological forms when growing in submerged culture, ranging from homogenously dispersed hyphae to compact, hyphal agglomerates known as pellets (Veiter et al. 2018). Each morphological class affects viability, productivity and performance in different ways. From a process control standpoint, pellets are favoured as rheology, gas–liquid mass transfer and mixing are facilitated. However, pellet morphology also leads to active and non-active zones within the pellet due to limitations in transport of substrates and products, especially oxygen (Dynesen and Nielsen (2003)). These zones also affect productivity, as production of penicillin is happening in the non-growing cytoplasm found in the pellet’s outer layer (Baumgartl et al. 1984). In turn, the pellet’s core exhibits hyphal degradation, a decline in viability and no productivity (Ehgartner et al. 2017a, b). Naturally, these variations across all morphological forms complicate viability estimation and by extension determination of growth rate, substrate uptake rates and yields.

Hence, a quantitative approach to assess viable biomass is of utmost importance. Determination of viability can be performed employing at-line chemical methods such as fluorescent staining or physical techniques using various sensors. Dielectric spectroscopy, infrared spectroscopy and fluorescence have been comprehensively studied in the scope of filamentous fungi (Ronnest et al. 2011; Ehgartner et al. 2017a, b). While these methods enable real-time measurement, they cannot take into account morphological aspects directly. In this respect, flow cytometry is a potent alternative. Biomass morphology can be classified according to size and form through analysis of light scatter signals (Dubelaar et al. 1999; Ehgartner et al. 2017a, b; Pekarsky et al. 2018). To assess viability, fluorescent staining is regularly used in flow cytometry coupled with fluorescence detectors (Langemann et al. 2016; Attfield et al. 2000; Pekarsky et al. 2018). For filamentous fungi such studies are scarce, mainly due to the large particle sizes of fungal biomass (Dubelaar et al. 1999). Recent studies encompass *Aspergillus niger* microcolonies and *Trichoderma* (de Bekker et al. 2011; Delgado-Ramos et al. 2014), but are lacking detailed morphological analysis. Specific applications of flow cytometry for morphological classification of *Penicillium chrysogenum* were recently published (Ehgartner et al. 2017a, b); however, they did not include viability assessment yet.

In this publication, we quantitatively employ flow cytometry to combine detailed morphological insights with viability assessment. The developed method is at-line and potentially online applicable, statistically sound due to the high number of measured particles, and can estimate viable layers in specific morphological classes, such as pellets and large hyphal agglomerates. Furthermore, we have verified our results with established state-of-the-art methods such as a plate reader method for viability assessment as well as confocal laser microscopy for determination of a viable pellet layer. In the following, these points will be discussed: (i) differentiation of viable biomass against complex media background, (ii) morphological analysis and assessment of viability, (iii) comparison of flow cytometry viability assessment with the state-of-the-art plate reader method, (iv) analysis of large element morphology and viable layer and (v) comparison of results from flow cytometry with confocal laser microscopy.

## Materials and methods

### Strain

Spore suspensions of the P-14 *P. chrysogenum* candidate strain for penicillin production descending from the P-2 *P. chrysogenum* candidate strain (American Type Culture Collection with the access number ATCC 48271) (Lein 1986) were provided by Sandoz GmbH (Kundl, Austria) and used for all experiments.

### Bioreactor cultivations

Three cultivations (FB1 and FB2) were performed in a Techfors-S bioreactor (Infors HT, Bottmingen, Switzerland) with a 10-l working volume. The batch was cultivated with an initial volume of 6.5 l in the first mentioned bioreactor and inoculated with 2 × 10^8^ spores/l. During batch phase pH was not controlled. The end of the batch was defined per default as an increase in pH of 0.5 by convention. After the batch, the broth was diluted with fed-batch medium (15% broth, 85% medium) and fed-batches were started with an initial volume of 6.5 l. Batch and fed-batch media were similar as described elsewhere (Posch and Herwig 2014).

During the fed-batch phase, pH was kept constant at 6.5 ± 0.1 by addition of 20% (*w*/*v*) KOH or 15% (*v*/v) H_2_SO_4_, respectively. pH was measured using a pH probe (Hamilton, Bonaduz, Switzerland). After additional 12-h nitrogen and phenoxyacetate feeds were started at constant rates (6.5 ml/h for nitrogen and 2 ml/h for phenoxyacetate). In the first 24 h of the fed-batch, 500 g/l glucose solution was fed at a constant rate of 1.01 ml/(l∙h). Afterwards, a three-times increase in feeding rate was carried out leading to a constant rate of 3 ml/(l/h).

The stirrer was equipped with three six-bladed Rushton turbine impellers, of which two were submersed and one was installed above the maximum liquid level for foam destruction. Fermentation temperature was kept at 25 °C via a cooling/heating jacket. Aeration was controlled at 1 vvm in batch and initial fed-batch with mass flow controllers (Vögtlin, Aesch, Switzerland). Dissolved oxygen concentration was measured using a dissolved oxygen probe (Hamilton, Bonaduz, Switzerland) and controlled between 40 and 90% during batch and between 40 and 60% during fed-batch, via adjustment of stirrer speed. The initial agitation conditions were 325 rpm stirring speed in batch and 500 rpm in fed-batch. CO_2_ and O_2_ concentrations in the off gas were analysed with an off-gas analyser (M. Müller AG, Switzerland).

Both cultivations were similarly conducted in a standard manner to generate biomass for method development. Only in FB2 was this strategy slightly altered: in order to measure a sudden viability decline, aeration was switched from air to N_2_ for FB2 at a process time of 160 h, which caused an immediate drop in dissolved oxygen concentration and CO_2_ concentration in the off gas.

### Flow cytometry

Samples from fed-batch cultivations were diluted 1:10 into phosphate-buffered saline (50 g/l of 2.65 g/l CaCl_2_ solution, 0.2 g/l KCl, 0.2 g/l KH_2_PO_4_, 0.1 g/l MgCl∙6 H_2_O, 8 g/l NaCl and 0.764 g/l Na_2_HPO_4_ + 2 H_2_O) and stained with propidium iodide (Sigma-Aldrich, St. Louis, Missouri/USA; 20 mM stock dissolved in DMSO ≥ 99.9%, diluted with phosphate-buffered saline to a final concentration of 20 μM). In order to study different viability stages, some samples were subjected to microwave treatment for 30 s at 940 W in a microwave oven. After incubating for 5 min, the sample was further stained with fluorescein diacetate (Sigma-Aldrich, St. Louis, Missouri, USA; stock solution of 5 g/l dissolved in acetone ≥ 99.9% to a final concentration of 5 mg/l). After incubation of 5 min, the sample was further diluted (1:100 in the same buffer) for flow cytometric analysis. Metabolic activity is shown by fluorescein diacetate (FDA) treatment resulting in green fluorescence through esterase activity. PI fluorescence is a result from DNA intercalation in cells with compromised membranes (Pekarsky et al. (2018)).

A CytoSense flow cytometer (CytoBuoy, Woerden, Netherlands) with two forward scatter (FSC), one sideward scatter (SSC) and two fluorescence channels (green, red) was used for particle analysis. The implemented laser had a wavelength of 488 nm. The configuration of the filter set was 515–562 ± 5 nm for the green fluorescence channel (FL-green, used for fluorescein diacetate) and 605–720 ± 5 nm for the red fluorescence channel (FL-red, used for propidium iodide). The device was equipped with a PixeLINK PL-B741 1.3MP monochrome camera for in flow image acquisition. For data treatment, the software CytoClus3 (CytoBuoy, Woerden, Netherlands) and a custom-programmed Matlab 2016b script (MathWorks, Natick, Massachusetts, USA) were used.

The CytoSense flow cytometer provides multiple data points per channel per particle. This signal shape is achieved for both scatter channels as well as green and red fluorescence channels (Dubelaar et al. 1999). These pulse shapes are the basis for multiple curve parameters (Ehgartner et al. 2017a, b). Except for length parameters in micrometres, all parameters are in arbitrary units, as the user can set sensitivity levels SSC and fluorescence detectors. Setting of sensitivity levels was aligned with plate reader viability assessment. The most relevant parameters for the here presented study are the following parameters: maximum (maximum of signal curve), total (area under curve), length (length of the signal), sample length (length of signal above trigger level) and fill factor (similarity of the curve to a block; 0–1; higher if block-shaped).

### At-line viability measurement via a plate reader

To investigate viability via propidium iodide (PI) staining, 200 μl of samples was diluted 1:5 with phosphate-buffered saline (PBS, see Ehgartner et al. 2017a, b). In addition, 1 ml of sample was diluted 1:5 with PBS and microwave treated by leaving it for 30 s at 940 W in a M510 microwave oven (Philips, Amsterdam, Netherlands). One millilitre of the microwave-treated sample was used for further investigation. In a next step, duplicates of all samples (including microwave treated and untreated samples) were centrifuged for 15 min at 500 min^−1^ and 50 g. Eight hundred microlitres of supernatant was removed, and 800 μl of PBS buffer was added. The pellet was resuspended and the washing step repeated. One hundred microlitres of the resuspended sample was pipetted into a microtiter well, and 1 μl of 200 μM PI solution (Sigma-Aldrich, St. Louis, Missouri/USA) was added. The PI was prepared by diluting a 20-mM PI stock solution in DMSO, 1:100 in PBS. After an incubation time of 20 min at room temperature in darkness, the measurement was performed in a Tecan well-plate reader (Tecan, Männedorf, Switzerland; ex./em. 535/600 nm). Each sample was measured six times simultaneously using 96-well plates. Viability is estimated according to Eq. 1:

1 $$ \mathrm{Viability}\ \left[\%\right]=\left(1-\frac{\mathrm{red}\ \mathrm{fluorescence}\ \mathrm{orginal}\ \mathrm{sample}}{\mathrm{red}\ \mathrm{fluorescence}\ \mathrm{in}\ \mathrm{microwaved}\ \mathrm{sample}\ }\right)\ast 100 $$

### Confocal laser fluorescence microscopy

Confocal fluorescence microscopy was used as a method to distinguish between viable and dead parts of the pellets. FDA was used to stain metabolically active, viable hyphae while PI was used to stain dead cells. Microscopic images of the pellets were taken using a confocal fluorescent microscope (TE2000-E, Nikon, Japan).

One hundred microlitres of the bioreactor sample was diluted with 900 μl of PBS buffer and then centrifuged for 2 min at 500 rpm at room temperature. Eight hundred microlitres of the supernatant was discarded and replaced with 800 μl of PBS buffer. Afterwards, 10 μl of 200 μM PI reagent (prepared from 20 mM stock solution by 1:100 dilution) was added and the sample was incubated for 10 min in the dark. Twenty microlitres of the sample was then applied on a cover slide, and the slide was then placed on the microscope table. After focusing, 2 μl of freshly prepared 50 mg/l FDA reagent (Sigma-Aldrich, St. Louis, Missouri, USA; prepared with PBS buffer from a stock solution of 5 g/l dissolved in acetone) was added and a cover slide was placed on the sample. Lasers and the respective detector systems (PI: ex. 543 nm, em. 580 nm; FDA: ex. 488 nm, em. 507 nm) were activated separately. The gain for the 507-nm channel was adjusted according to FDA-related fluorescence intensity increase. The pellet was focused with the maximum intension of the PI stained area as criterion. Pictures were taken for at least 10 pellets per sample.

## Results

### Differentiation of viable biomass against complex media background

Based on initial measurements of the fed-batch medium with and without cells, a distinction of fungal cells from the media background was possible. Particles exceeding a green fluorescence signal of 500 were classified as viable cells. Within previously set gates, viable cells and dead cells are easily differentiated from the media background as displayed in Fig. S1.

In principle, this differentiation is also possible without the use of fluorescent staining. However, results are negatively influenced by the media particle content (as demonstrated in Fig. S2) making a sharp distinction impossible. Naturally, fluorescence intensity of unstained biomass is 10–50 times lower as well, which further complicates differentiation.

### Morphological analysis and assessment of viability

Scatter plots from viable cell data were generated, and gates were set for morphological classification as previously described by Ehgartner et al. (2017a, b). This method enables classification according to the following forms: hyphae, small clumps, large clumps and pellets. Summarizing, gate setting is based on particle size in combination with SSC total. For differentiation between large clumps and pellets, saturation of FSC signals, respectively FSC fill factor values are considered. Exact definitions on the differentiation between morphological fractions are provided in Table 1. In the following, large clumps and pellets will frequently be characterized in a combined fashion as “large elements”.

**Table 1:** Measurement errors of common parameters determined by previously described methods

Parameter	Plate reader staining method		Flow cytometry		Confocal laser microscopy	
	%	Absolute	%	Absolute	%	Absolute
Viability overall	7	–	5	–		
Viability—hyphae	2	–	4	–		
Small clumps	2	–	3	–		
Large clumps	–	–	6	–		
Pellets	–	–	6	–		
Viable layer large elements			13	6 μm	–	–
Viable layer pellets			7	2 μm	11	3 μm

For large clumps and pellets, the parameter “core compactness” representing the density of the pellet core can be derived from the following Eq. 2.:

2 $$ {\mathrm{Compactness}}_{\mathrm{core}}\ \left[-\right]=\frac{\mathrm{Length}\ \mathrm{of}\ \mathrm{saturated}\ \mathrm{FSC}\ \mathrm{signal}\ \mathrm{in}\ \mathrm{pellet}\ \mathrm{core}\ \left[\upmu \mathrm{m}\right]}{\mathrm{Core}\ \mathrm{diameter}\ \left[\upmu \mathrm{m}\right]} $$

Similar knowledge can be obtained from the analysis of SSC signal length in combination with particle size, hereafter termed “Compactness according to SSC” and calculated according to Eq. 3.:

3 $$ {\mathrm{Compactness}}_{\mathrm{SSC}}\ \left[-\right]=\frac{\mathrm{Length}\ \mathrm{of}\ \mathrm{SSC}\ \mathrm{signal}\ \left[\upmu \mathrm{m}\right]}{\mathrm{Particle}\ \mathrm{diameter}\ \left[\upmu \mathrm{m}\right]} $$

**Fig. 1:**
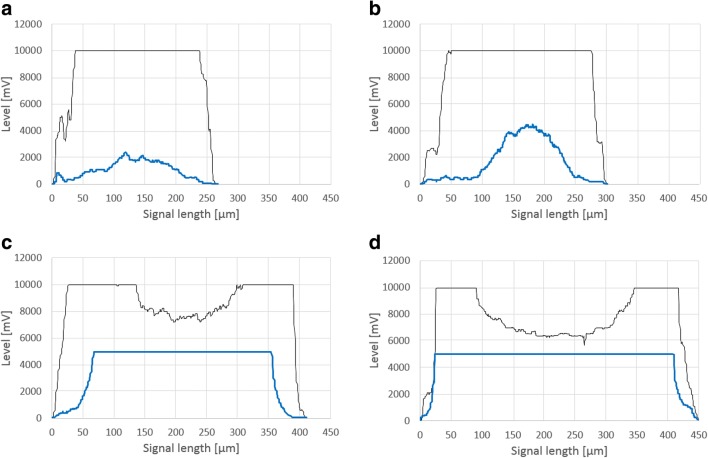
Pulse shape profiles of several pellets. Black line for FSC signal, blue line for SSC signal. Demonstration of compactness calculation according to Eqs. 1 and 2. Low diameter and low SSC signal; saturated FSC signal equals high core compactness (**a**). Low diameter and increased SSC signal, and saturated FSC signal (**b**). Saturated SSC signal and pellet breakage according to FSC signals at elevated pellet diameters equals low core compactness and high compactness _SSC_ (**c**, **d**)

Images of several pellet signal pulse shapes including FSC and SSC are displayed in Fig. 1. These images are representative of several process stages in FB2. Figure 1a shows a pellet with rather low compactness according to the SSC signals; the pellet depicted in Fig. 1b displays increased compactness. In Fig. 1c, d, breakage can be observed from FSC signals in the pellet’s core. Both of these degrading pellets display high compactness according to SSC signals. This is in accordance with literature, as highly compact pellets (as indicated by SSC signal saturation) exhibit hyphal degradation and a decline in viability in the pellet’s core (Ehgartner et al. 2017a, b).

**Fig. 2:**
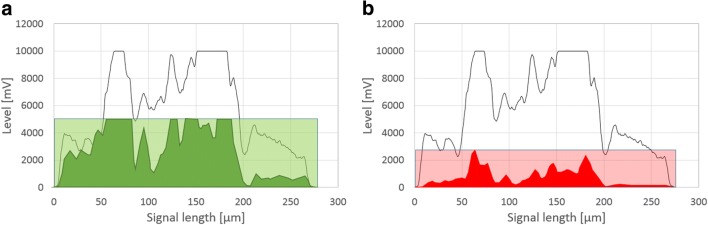
Pulse-shape signal profiles for assessment of viability according to Eqs. 3 and 4. Light green area indicates block shape of green fluorescence for fill factor calculation; dark green are indicates area under green fluorescence curve (**a**). Light red area indicates block shape of red fluorescence for fill factor calculation; dark red area indicates area under red fluorescence curve (**b**)

Assessment of viability is based on individual particle pulse shapes, as demonstrated in Fig. 2. In order to estimate contributions of both fluorescence signals to viability, the particle parameter “fluorescence fill factor” (FL_FF_) (see Eq. 4) for green and red fluorescence was derived from fluorescence pulse shapes according to the following equation:

4 $$ {\mathrm{FL}}_{\mathrm{FF}}\ \left[-\right]=\frac{\mathrm{Area}\ \mathrm{under}\ \mathrm{FL}\ \mathrm{curve}\ \left[\mathrm{mV}\ast \upmu \mathrm{m}\right]}{\mathrm{Area}\ \mathrm{under}\ \mathrm{block}-\mathrm{shaped}\ \mathrm{FL}\ \mathrm{curve}\ \left[\mathrm{mV}\ast \upmu \mathrm{m}\right]\ } $$

Individual particle viability can be estimated according to Eq. 5.

5 $$ \mathrm{Individual}\ \mathrm{particle}\ \mathrm{viability}\ \left[\%\right]=\frac{\mathrm{Area}\ \mathrm{under}\ {\mathrm{FL}}_{\mathrm{green}}\ \mathrm{curve}/\mathrm{Area}\ {\mathrm{FL}}_{\mathrm{red}}\ \mathrm{curve}}{1+\mathrm{Area}\ \mathrm{under}\ {\mathrm{FL}}_{\mathrm{green}}\ \mathrm{curve}/\mathrm{Area}\ {\mathrm{FL}}_{\mathrm{red}}\ \mathrm{curve}}\ast 100 $$

$$ \mathrm{Area}\ \mathrm{under}\ \mathrm{curve}\ \left[\mathrm{mV}\ast \upmu \mathrm{m}\right] $$

**Fig. 3:**
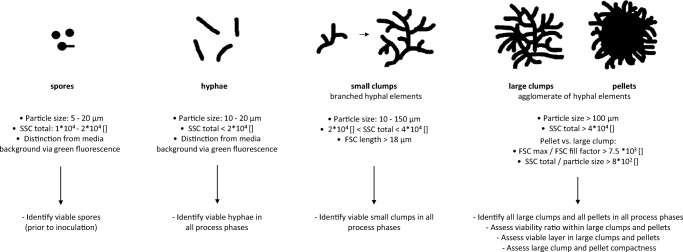
Information gain from individual morphological classes with definition of morphological classes according to flow cytometry

In order to assess overall viability, the mean value of all particle viabilities in a certain morphological class or in all morphological classes can be calculated. Figure 3 provides an overview on the information to be obtained from individual morphological classes. Viable spores can be quantified in pre-culture media prior to inoculation (as described by Ehgartner et al. 2016). Viable hyphae and small clumps are detectable though staining in all process phases which were previously described in “Materials and methods” under bioreactor cultivations. Large clumps and pellets can be identified in particle-free fed-batch media without staining due to auto-fluorescence (see Fig. S2). Overall different viabilities can also be subdivided into the viability of different morphological classes and vice versa (see Fig. S3). Further information obtained from large elements comprises the viability ratio within biomass particles, the assessment of the viable layer and the parameter “compactness” (as described by Ehgartner et al. 2017a, b).

**Fig. 4:**
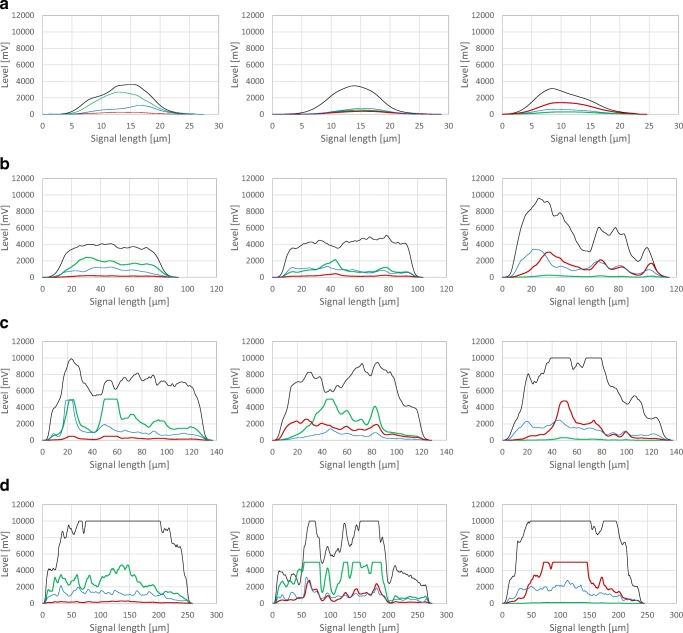
Pulse shape profiles of several morphological classes. Hyphae (**a**), small clumps (**b**), large clumps (**c**), and pellets (**d**). High viability (left column), reduced viability (middle column), low viability in microwaved samples (right column). FSC signal (black line), SSC signal (blue line), FL-green signal (green line), and FL-red signal (red line). Lines top to bottom: increases in FSC and SSC signal indicate rise in particle size and compactness due to morphological class defined in Fig. 3. Columns left to right: a decline in viability is seen with increasing FL-red signals and decreasing FL-green signals

Typical pulse shape profiles are specifically obtainable by the CytoSense flow cytometer. They depict various viability states for each morphological class as displayed in Fig. 4 for all four morphological classes. When analysing large structures, saturation of several signals was observed (see Fig. 4d). This can be expected, as the system needs to be capable of measuring fluorescence in a wide range of particle sizes. Consequently, a compromise between the detection of low fluorescence due to high sensitivity settings and potential loss of information in large particles needs to be found. High green fluorescence also led to a “bleeding” effect into the red fluorescence channel. However, this phenomenon was not prominent in microwaved negative control samples. Here, an increase in red fluorescence is not related to green fluorescence and indicated viability-declined agglomerates, as PI cannot cross the membrane of healthy cells (Soderstrom (1977); Pekarsky et al. 2018).

### Comparing flow cytometry viability assessment with the state-of-the-art plate reader method

**Fig. 5:**
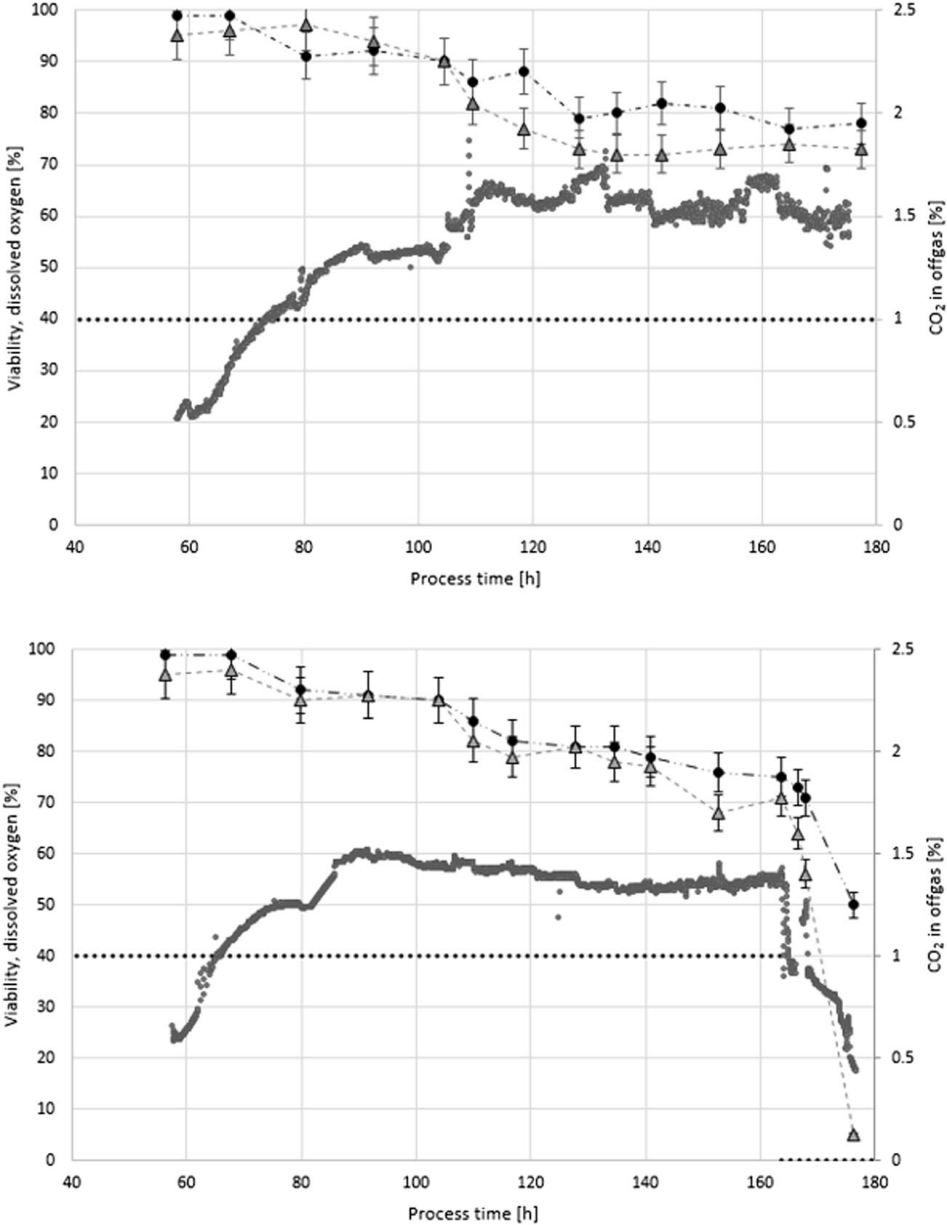
Comparison of flow cytometry viability assessment with the state-of-the-art plate reader method. FB1 (top), FB2 (bottom). Plate reader (black). Flow cytometry (grey). Dissolved oxygen set point (dotted line), CO_2_ in off-gas (grey points). Dissolved oxygen set point for FB2 was decreased to 0% at process time 160 h to cause drop in viability

To compare viability assessment from flow cytometry data with a previously established at-line viability measurement via plate reader, hereinafter called state-of-the-art method (Ehgartner et al. 2017a, b), two fed-batch cultivations were performed and samples were measured employing both at-line methods. Viability assessment data from cultivations FB1 and FB2 are displayed in Fig. 5. For standard process conditions, both methods show similar results. In FB2, a deliberate process deviation through a downregulation of dissolved oxygen at process time 160 h (see Fig. 5) was introduced which was immediately followed by a drop in off-gas CO_2_ signals (see Fig. 5). Only the flow cytometry method immediately registered the impact on viability assessment. After 3 h, a 10% decrease in viability is depicted, after 10-h overall viability is estimated at only 5%. This is consistent with off-gas and productivity data (data not shown). The viability drop is also observable in the plate-reader viability assessment, but less prominent and observable only in a delayed manner. This can be explained by the nature of the stain used in the plate-reader method: with PI, an immediate drop in metabolic activity as found through the use of FDA cannot be detected as PI can indicate viability loss only through DNA intercalation in cells with compromised membranes (Soderstrom 1977; Pekarsky et al. 2018).

### Analysis of large element morphology and viable layer

A more detailed assessment of viability is possible in large elements due to signal length and associated information. On the one hand, the viable layer can be obtained from a comparison of green fluorescence derived from FDA staining with the particle’s FSC signal. In combination with the known particle size, the viable layer (vl) can be estimated according to Eq. 6. On the other hand, red fluorescence from PI staining indicates viability loss. The pellet’s centre should harbour an increased amount of degenerated hyphal structures. Red fluorescence signals behave accordingly and display strong values or even saturation, usually in the core area. Consequently, vl can be defined via the setting of a threshold value (see Eq. 7) which indicates degradation when exceeded. This approach comes with the disadvantage that setting of the threshold is arbitrary and therefore needs to be aligned with data from other methods (e.g. confocal laser microscopy, see next section). In both approaches, the factor 0.5 is added to calculate vl according to radius and not diameter when assuming the pellet to be a sphere/circle.

6 $$ \mathrm{viable}\ \mathrm{layer}\ \mathrm{vl}\ \left[\upmu \mathrm{m}\right]=\frac{\mathrm{Area}\ \mathrm{under}\ \mathrm{FLG}\ \left[\mathrm{mV}\ast \upmu \mathrm{m}\right]\ }{\mathrm{Area}\ \mathrm{under}\ \mathrm{FSC}\ \left[\mathrm{mV}\ast \upmu \mathrm{m}\right]}\ast \mathrm{average}\ \mathrm{size}\ \left[\upmu \mathrm{m}\right]\ast 0.5 $$

7 $$ \mathrm{viable}\ \mathrm{layer}\ \mathrm{vl}\ \left[\upmu \mathrm{m}\right]=0.5\ast \left(1-\mathrm{Length}\ \mathrm{of}\ \mathrm{FLR}>\mathrm{threshold}\ \left[\upmu \mathrm{m}\right]\right) $$

**Fig. 6:**
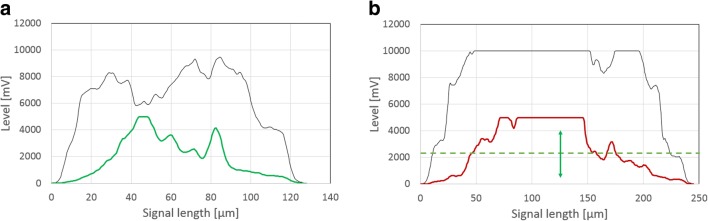
Approaches for determination of viable layer. Assessment via FL-G (**a**) or FL-R signal (**b**)

Both approaches are outlined in Fig. 6. Assessment according to Eq. 5 is more reliable, as effects of particle size on fluorescence signals across all particles cannot be considered by the setting of a fixed threshold.

### Comparing results from flow cytometry with confocal laser microscopy

**Fig. 7:**
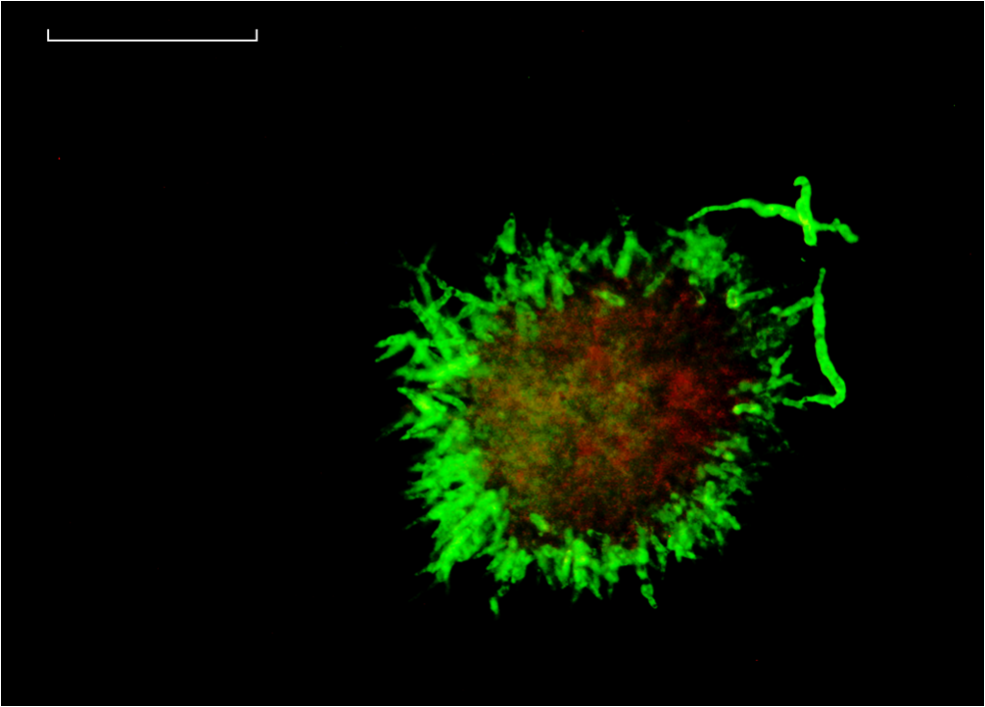
Confocal microscopy of pellet with enhanced contrast. The sample was taken from FB1 at a process time of 80 h after transfer. Green fluorescence from FDA staining represents viable pellet layer, red fluorescence from PI staining in pellet core. White line = 50 μm

Samples from FB1 were analysed to compare both methods for determination of a viable layer. Exemplary pellet analysis using confocal layer microscopy employing FDA and PI staining is shown in Fig. 7. A three-dimensional structure of viable regions can be estimated from this image, as red fluorescence harboured in the centre is partly overlaid by viable hyphal entanglements. Assuming a simplified two-dimensional cross section, a green fluorescent viable layer located around the outer hairy region is clearly distinguishable from the core, which exhibits degradation.

**Fig. 8:**
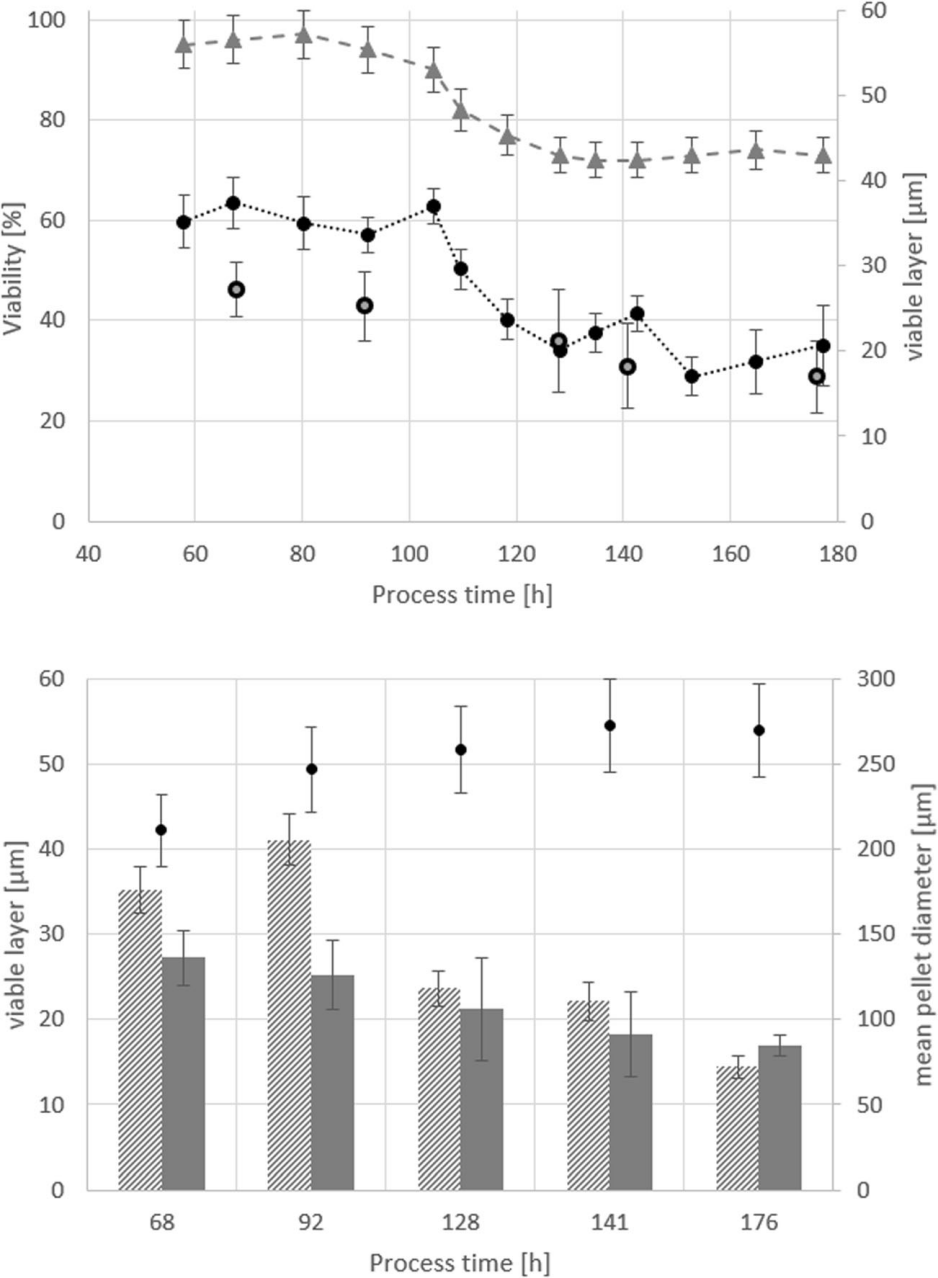
Comparison of confocal microscopy and flow cytometry, both methods employed to determine the viable layer in large elements taken from FB1 samples. Top: viability from flow cytometry (grey triangles), viable layer from flow cytometry (black circles) and viable layer from confocal microscopy (grey squares). Bottom: viable layer determined via flow cytometry (patterned bars), viable layer determined via confocal microscopy (grey bars) and average pellet diameter (black dots). Standard deviation in confocal microscopy calculated from at least 6 pellets analysed per sample

After confocal analysis of at least six pellets per sample, the average viable pellet layer in five different samples was determined and compared to flow cytometry results as depicted in Fig. 8. Both methods displayed similar values, apart from some deviations in the earlier process phases. In all samples, the viable layer was determined at roughly one-third of the whole pellet radius, which is in agreement with literature (Posch et al. 2012; Baumgartl et al. 1984; Nielsen et al. 1995; Justen et al. 1998). The mean pellet diameter in FB1 was 251.5 ± 25 μm.

## Discussion

### Advantages, disadvantages and comparability to other methods

Within this contribution, we present a novel combination of morphological analysis and viability assessment based on flow cytometry. This signifies a faster alternative to image analysis via microscopy and more statistical reliability due to the large number of particles being measured in sort time spans (as previously established by Ehgartner et al. 2017a, b). In addition, enhanced insight into viability is generated simultaneously through fluorescent staining: Overall viability, viability of morphological classes and the viable layer of large elements can be determined. This viability data is enhanced by morphological parameters like pellet and large element compactness.

To verify this technique, results were compared to data from respective state-of-the-art methods, namely at-line viability measurement via plate-reader for overall viability and confocal laser microscopy for determination of the viable pellet layer. To generate sufficient amounts of biomass with diverse morphology and viability states, bioreactor cultivations in fed-batch mode were conducted and extensively sampled. Each sample was subjected to flow cytometry and plate-reader viability measurement. For determination of overall viability, the flow cytometry method was superior as the effects of a sudden drop of dissolved oxygen were registered more reliably. A selection of samples from FB1 was also analysed using confocal laser microscopy to determine viable layers across pellets. Results of the flow cytometry method were in accordance with reference measurements. Furthermore, the method was applicable in complex media with high particle background.

The main distinguishing feature of the flow cytometry method is that viability in different morphological classes can be determined, even down to individual particles. Other methods generally only provide an overview on viability. This is especially useful in later process stages: small hyphal elements tend to be viable, while degradation in larger agglomerates and pellets is observable over time. Such large elements can be analysed in detail; thereby, viable and non-viable biomass sections are identified and quantified over each particle. However, a diverse morphology is a challenging thing and needs to be addressed: to guarantee comparable information content across all process phases, a compromise in fluorescence detector sensitivity settings must be found for individual strain/media combinations: in early process phases, detectors must be sensitive enough to detect viable biomass; in later stages, signal saturation needs to be avoided when possible. Furthermore, it should be noted that fluorescence spectral overlap might result in misleading signals. This is especially true for large elements harbouring considerable green fluorescence from FDA, which can also be registered by the red fluorescence detector as a misleading artefact (Bagwell and Adams 1993). Consequently, the ratio between red and green fluorescence needs to be checked regularly. Deviations in this ratio occur due to saturation effects from green fluorescence signals and spectral overlap or might indicate viability decline.

Disadvantages also include size-exclusion effects: due to the large size and compact nature of fungal pellets, they might be excluded at the opening of the sampling tube. As a result, small elements are generally over-represented while more information can be obtained from the evaluation of large elements. If the measurement goal is characterization of large elements, a simple solution to the size-exclusion issue would be to increase measurement times or set trigger factors in the software according to particle size. However, a representative overview on morphology respecting all size classes of morphology is more challenging. Depending on the fungal species and/or strain to be analysed, certain adjustments of the sampling tube could be considered, like a wider tubing or a cone at the end of the sampling tube.

### Applicability of the method

We envision this method to be a further milestone in the at-line characterization of complex fungal biomass (with a clear potential for online application through automated sampling systems) in process development and routine manufacturing processes. Based upon previous method development (as published by Ehgartner et al. 2017a, b), we enhanced morphological classification to analyse viability across all morphological forms with a special emphasis on the pellet’s viable layer. As a result, we are now able to combine morphological analysis with viability assessment in an at-line environment with potential online applicability through the use of automated sampling and sample processing. For this purpose, sampling, dilution and addition of fluorescent dyes needs to be performed in a modular process analytical (PAT) system with a flow cytometer connected.

We are confident that this method can shed a light on the complex and extensively researched relationship between fungal morphology, viability and productivity (Veiter et al. 2018; Wucherpfennig et al. 2011; Krull et al. 2013). While this method was developed for *P. chrysogenum*, we see the possibility to broaden applicability towards other filamentous fungi and by extent further agglomerate forming organisms such as yeast (Pekarsky et al. 2018).

## Electronic supplementary material

ESM 1 (PDF 198 kb)
