# Supplementary material for: The filamentous fungus Penicillium chrysogenum analysed via flow cytometry—a fast and statistically sound insight into morphology and viability
Source: Appl Microbiol Biotechnol. 2019 Jun 19;103(16):6725–35. doi: 10.1007/s00253-019-09943-4 (PMC6667401; doi:10.1007/s00253-019-09943-4)
Supplement: Supplementary file 1 — (PDF 198 kb) [file 253_2019_9943_MOESM1_ESM.pdf]

Supplementary Material for

The filamentous fungus *Penicillium chrysogenum* analysed via flow cytometry - a fast and statistically sound insight into morphology and viability

Lukas Veiter[1,2] and Christoph Herwig*[,1,2]

*to whom the correspondence should be addressed to

[1] CD Laboratory on Mechanistic and Physiological Methods for Improved Bioprocesses, Vienna University of Technology, Gumpendorferstrasse 1a/166, 1060 Vienna, Austria

[2] Research Area Biochemical Engineering, Institute of Chemical, Environmental and Bioscience Engineering, Vienna University of Technology, Gumpendorferstrasse 1a/166, 1060 Vienna, Austria

*Corresponding author:

christoph.herwig@tuwien.ac.at; Tel (Office): +43 1 58801 166400

Gumpendorferstrasse 1a / 166-4

1060 Wien, Austria

Journal: “Applied Microbiology and Biotechnology”

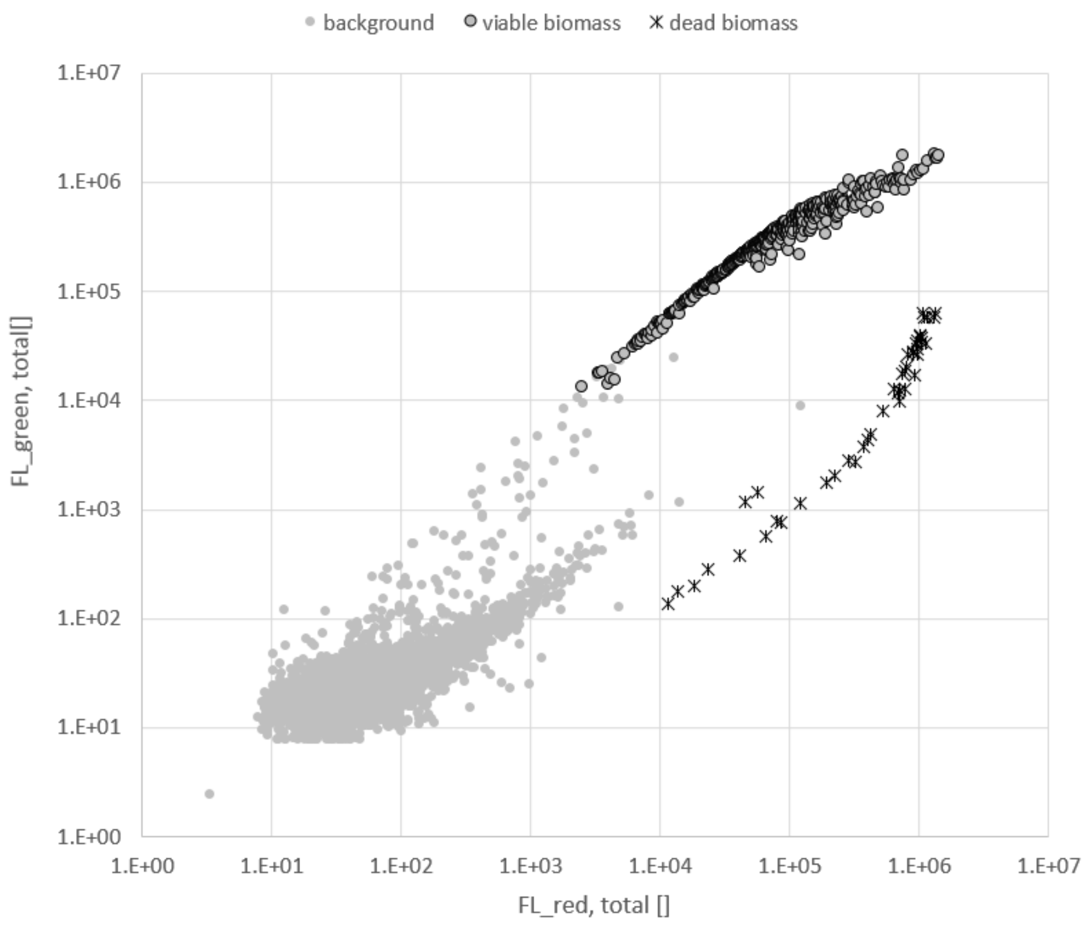

Figure S1. Sample from FB2 fed-batch phase 80h after transfer with overall viability of 90 % measured via flow cytometry and confirmed by plate reader staining measurements**.** Differentiation between complex media background, viable and dead biomass through gate setting employing fluorescent staining with FDA/PI. Viable biomass (framed circles), dead biomass (framed triangles) and background (grey circles).

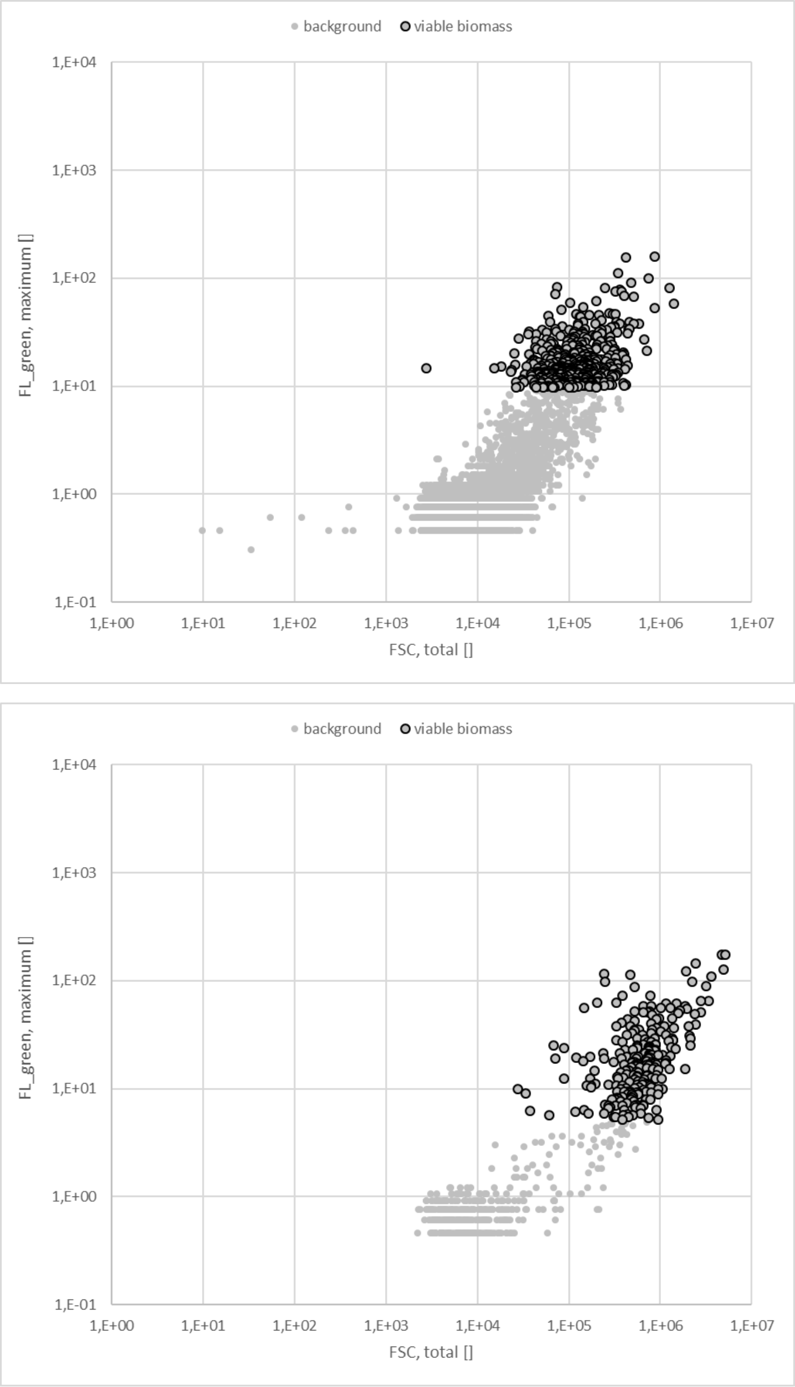

Figure S2. Comparison of biomass differentiation in different media without the use of fluorescent staining. High media particle content (top), low media particle content (bottom).

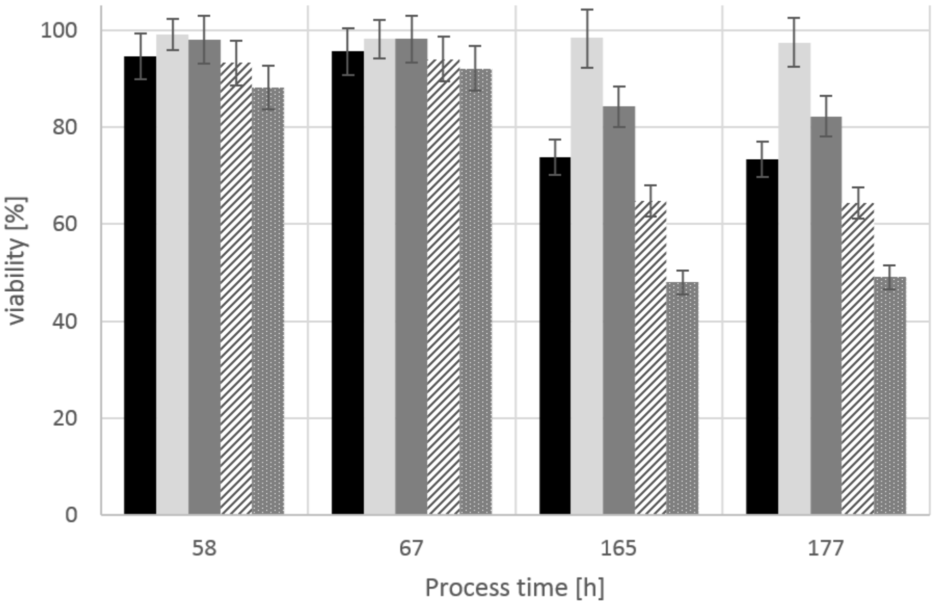

Figure S3. Viability determination via flow cytometry. Overall viability (black bars) at different process times subdivided into different morphological classes: hyphae (light grey bars), small clumps (dark grey bars), large clumps (patterned bars), pellets (dotted bars). Data from FB1 displaying beginning and end of fed-batch cultivation phase.
